# Supplementary material for: HDAC6 inhibition reverses axonal transport defects in motor neurons derived from FUS-ALS patients
Source: Nat Commun. 2017 Oct 11;8:861. doi: 10.1038/s41467-017-00911-y (PMC5636840; doi:10.1038/s41467-017-00911-y)
Supplement: Supplementary file 1 — Supplementary Information [file 41467_2017_911_MOESM1_ESM.pdf]

Description of Supplementary Files

File Name: Supplementary Information

Description: Supplementary figures, supplementary tables

File Name: Peer Review File

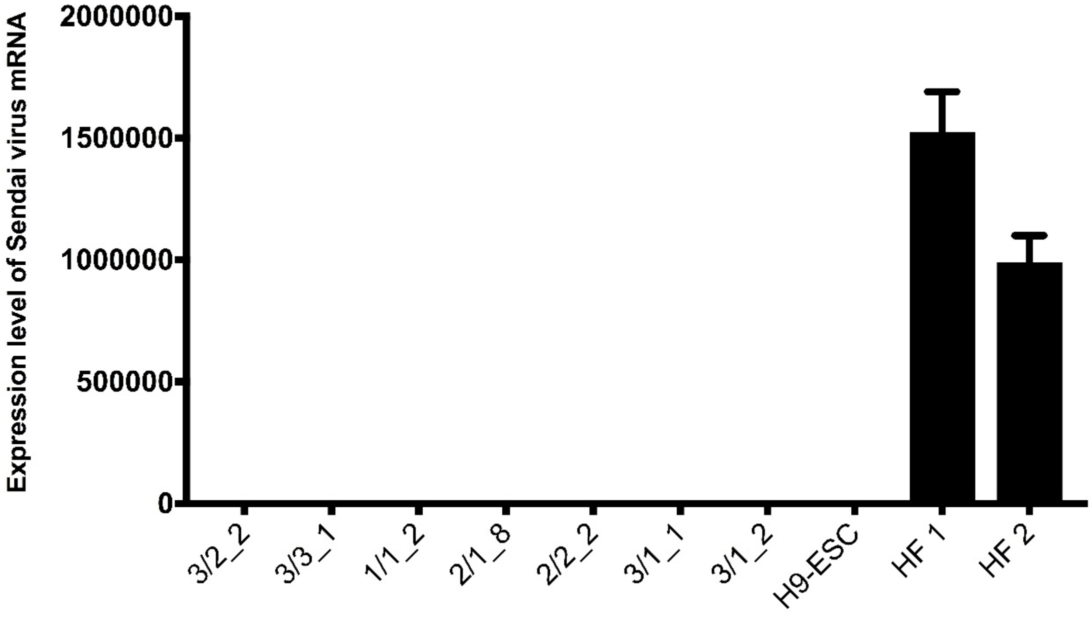

Supplementary Figure 1: Sendai virus detection in iPSCs

qPCR showing mRNA expression levels of Sendai virus in ALS iPSCs (different iPSC lines marked with numbers as shown in Table S1), hESCs (H9 embryonic stem cell line; negative control), fibroblasts 5 days after Sendai virus transfection (P1, P2; positive control of Sendai virus transfection from different cell culture dishes).

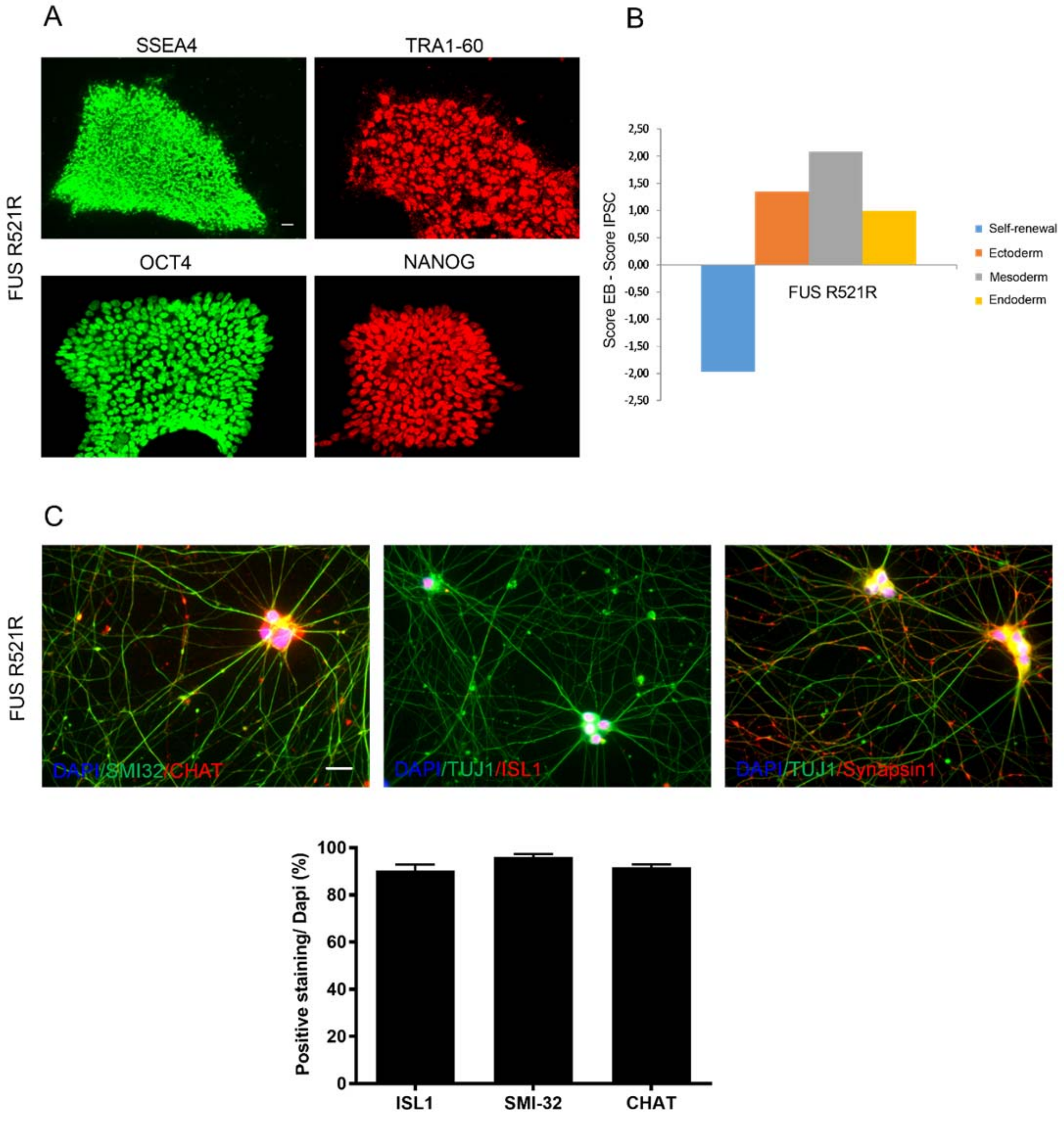

Supplementary Figure 2: iPSC pluripotency and MN differentiation capability of the isogenic control line

(A) Immunocytochemical staining of the isogenic control iPSCs (R521R) for the pluripotency markers: SSEA4, Tra1-60, Oct-4 and Nanog.

(B) Embryonic body formation experiment showing the presence of the three germ layer markers using qPCR.

(C) Staining with different MN markers (Isl1, Chat, SMI-32, Synapsin1) at day 38 of cells differentiated from the isogenic control iPSC line and quantification of ISL1-positive cells, SMI-32-positive cells and CHAT-positive cells expressed relative to the total number of DAPI labeled cells. N=10 images per line. Data are represented as mean ± SEM. Scale bar = 20µm.

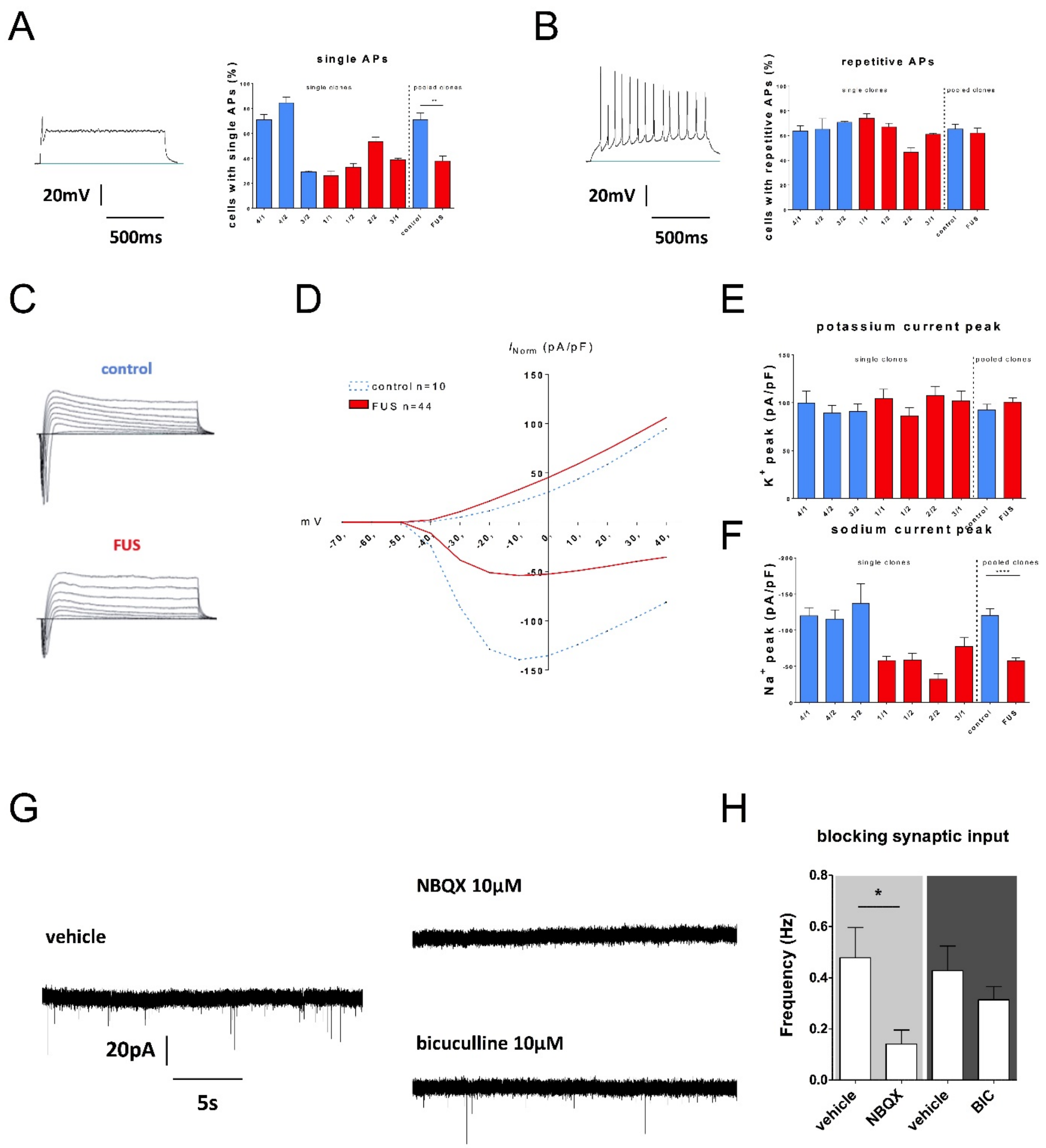

Supplementary Figure 3: Electrophysiological recordings of iPSC-derived MNs

(A-B) Representative current clamp recordings during ramp depolarization and quantification of single (A, n=110 and n=67 for control and patients, respectively; Mann-Whitney test, **P values is 0.01) or repetitive (B, n=110 and n=67 for control and n=67 for control and patients respectively) action potentials (APs) in mutant FUS expressing MNs and controls indicating that both are similar.

(C-F) Voltage dependent Na^+ and K^+ currents elicited upon stepwise depolarization in increments of 10 mV from a holding potential of -70 mV to 40 mV (C, D, n=10 and n=44 for control and patients, respectively). The normalized maximal Na^+ amplitudes (F, n=10 and n=44 for control and patients, respectively; Mann-Whitney test, ****P values is 0.0001) are

significantly lower in mutant FUS patients-derived motor neurons, but K^+ amplitudes (E, n=110 and n=67 for control and patients, respectively) showed no change.

(G-H) The recorded post synaptic currents (PSCs) are blocked by the glutamatergic AMPA receptor antagonist NBQX (10µM), but not with the $GABA_A$ receptor antagonist bicuculline (10 µM). n=7 for vehicle (left) and NBQX; n=5 for vehicle(right) and BIC. Mann-Whitney test,*P values is 0.05.

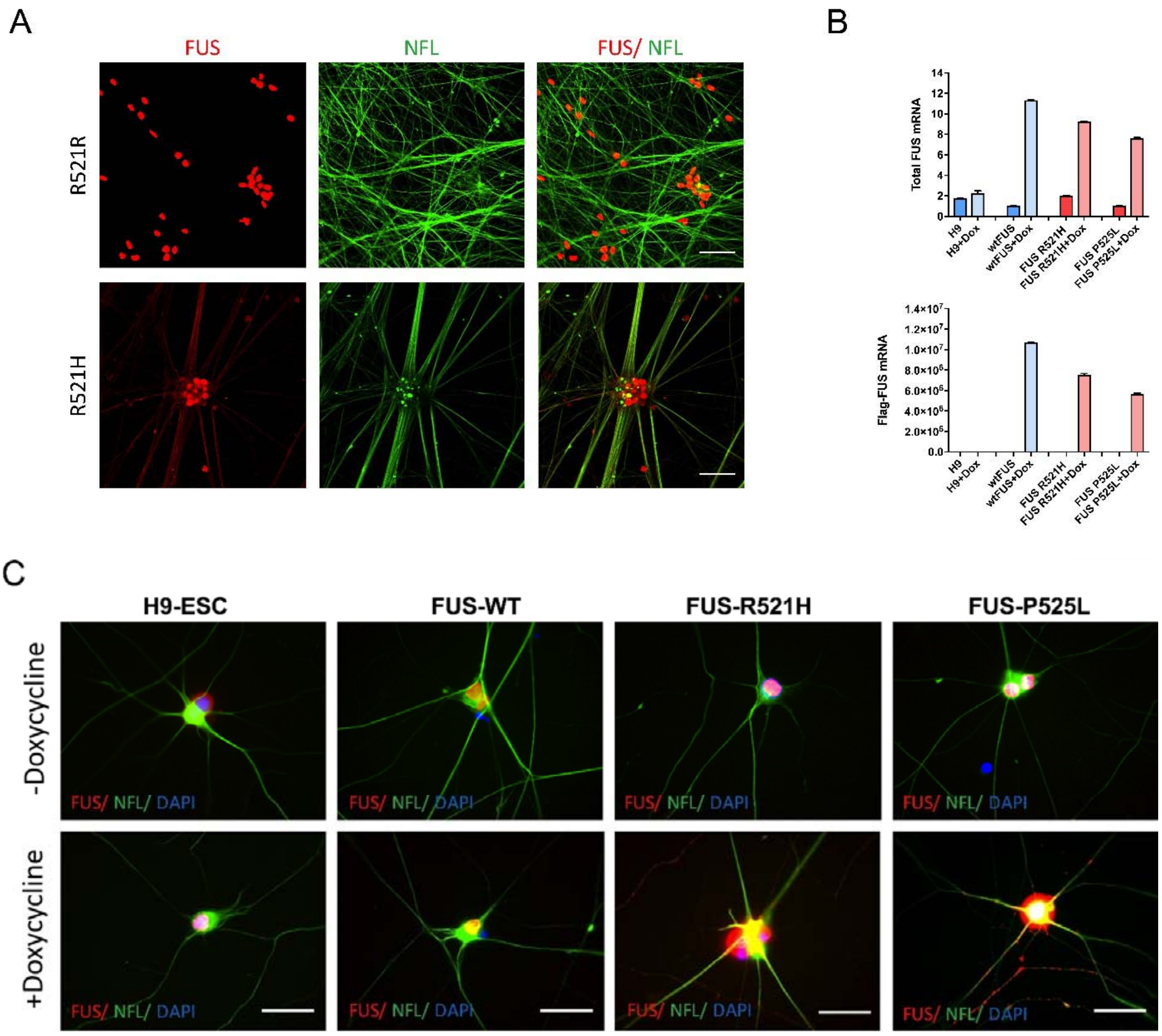

Supplementary Figure 4: Expression of FUS in iPSCs and hESC

(A) Immunostainning of FUS (red) and Neurofilament light chain (green) in the R521H mutant line (2/2) and in the isogenic control (R521R). Scale bar = 50 µm.

(B) Quantification using qPCR of the total amount of FUS or knocked-in FUS (with 3×flag) mRNA in H9-hESC and in the different FUS overexpressing hESC lines before and after adding doxycycline (1µg/ml) from day 17 until day 38 of MN differentiation.

(C) Immunostaining of FUS and Neurofilament light chain in inducible hESC lines. Scale bar = 20 µm.

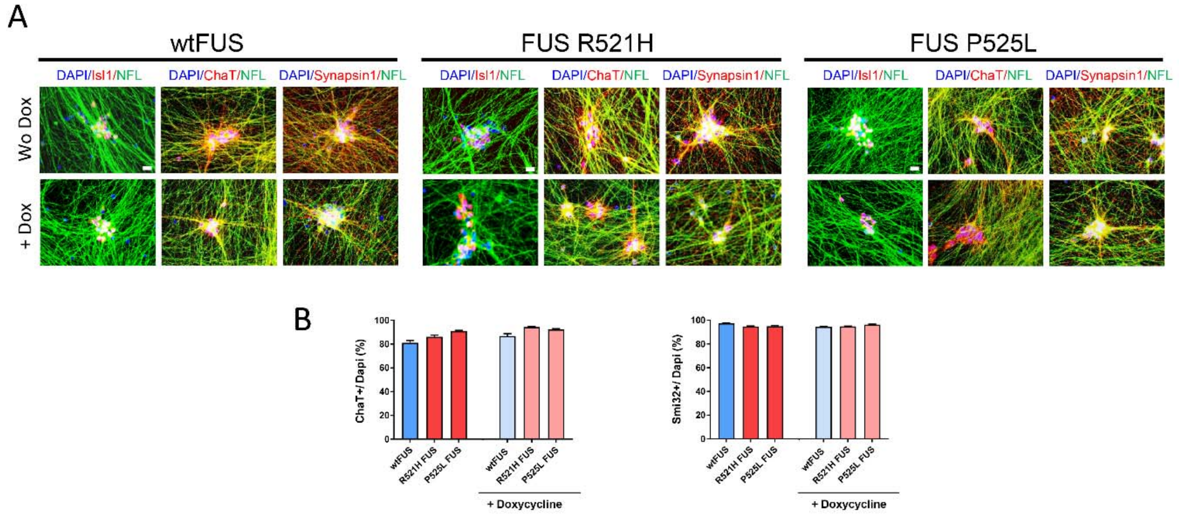

Supplementary Figure 5: No effect of FUS expression on differentiation of hESCs into MNs

(A) Different hESCs containing inducible constructs expressing wild type (wt) FUS or two different mutant FUS constructs (R521H and P525L) integrated into the *AAVS1* locus were differentiated into MNs and were stained for choline acetyltransferase (ChaT), Isl1, NFL and Synapsin1in combination with a DAPI staining. In the '+Dox' condition, doxycycline (1µg/ml) was added from day 17 until day 38.

(B) Quantification of relative number of cells staining positive for the MN markers ChaT (left) and SMI32 (right) relative to the total number of DAPI-positive cells in the absence and presence of doxycycline. No effect of FUS expression was observed. N=10 images per condition, scale bar= 20 µm.

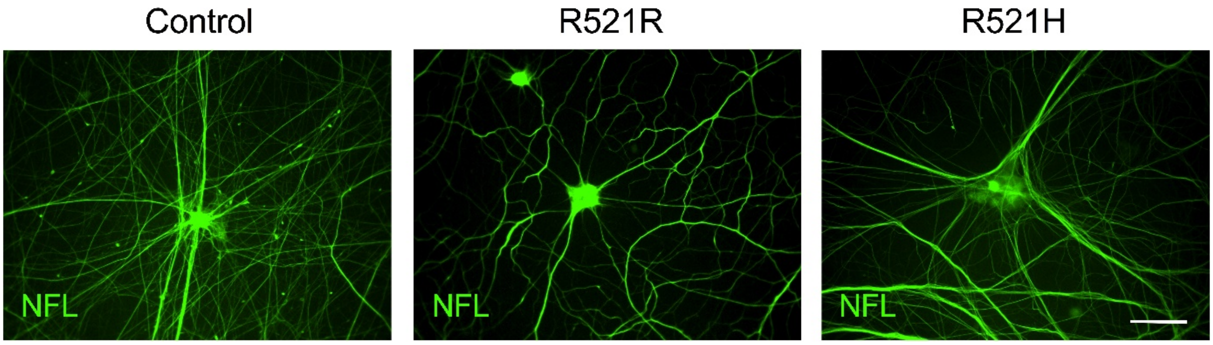

Supplementary Figure 6: Neurofilament light chain (NFL) staining of MNs differentiated from iPSCs.

Immunostaining of NFL in MNs derived from iPSCs of a healthy control (3/2), an isogenic control (R521R) and a patient (2/2) line. MNs were stained at the 4th week of differentiation. Scale bar = 20 µm.

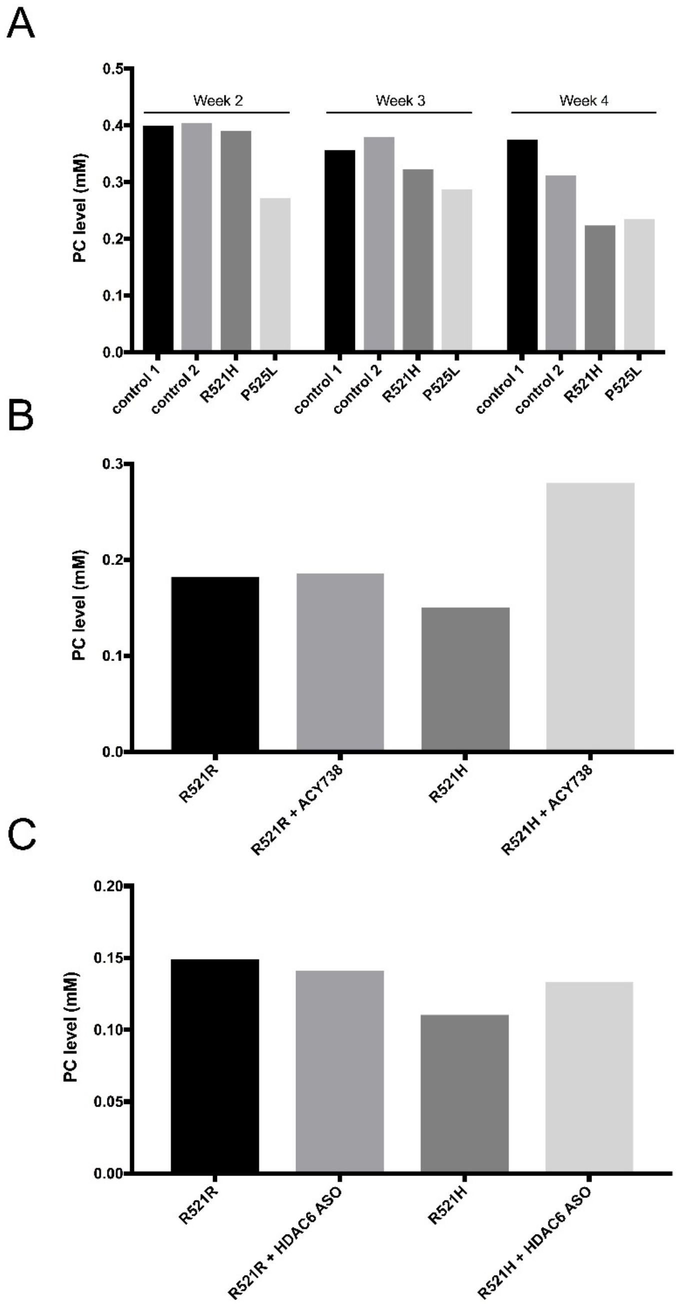

Supplementary Figure 7: ELISA detection of phosphatidylcholine levels as a function of differentiation

(A) Phosphatidylcholine levels in culture media of MNs show a decreased trend in patient-derived cells. Medium samples were taken for ELISA assays after two days on the culture. Control lines (control 1: 3/2; control 2: 3/3) are shown in blue and patient lines (R521H: 2/2; P525L: 3/1-2) in red.

(B) Phosphatidylcholine level in culture medium of MNs derived from patient iPSCs and isogenic controls with or without an overnight treatment with ACY-738 (1μM).

(C) Phosphatidylcholine level in cultured medium of patient and isogenic control MNs with or without HDAC6 knock down using an ASO.

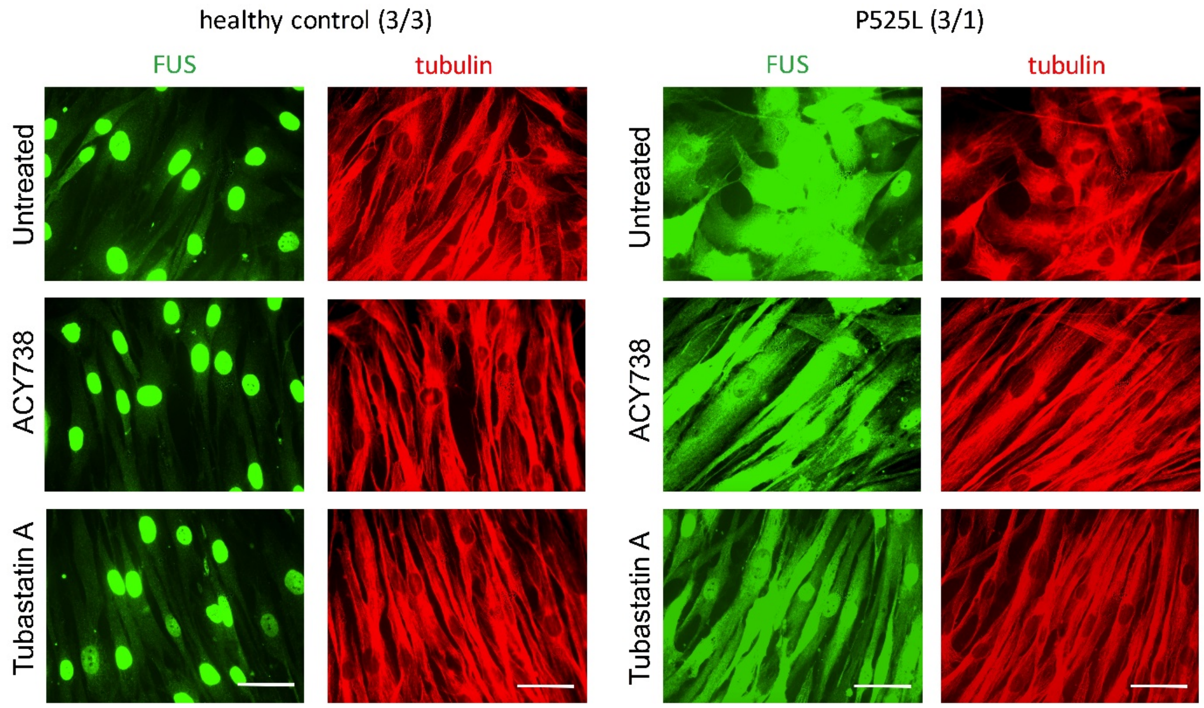

Supplementary Figure 8: No effect of HDAC6 inhibition on FUS localization in human fibroblasts

Immunostaining for FUS in fibroblasts (3/1 patient carrying the P525L mutation and 3/3 is a healthy control) with or without a treatment with ACY-738 (1µM) or Tubastatin A (1µM). Scale bar= 40 µm.

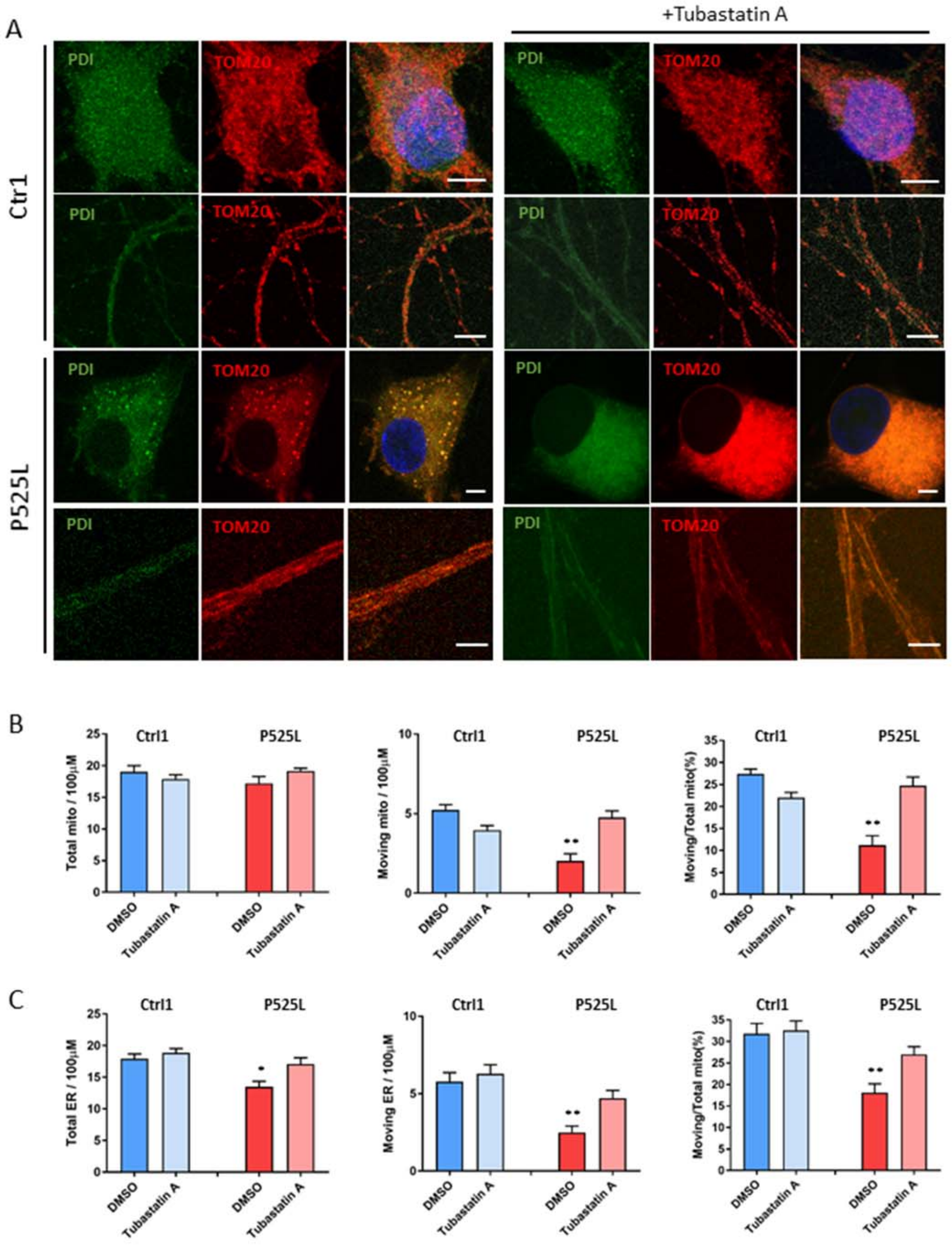

Supplementary Figure 9: Restoration of ER-mitochondrial overlay and axonal transport by Tubastatin A treatment

(A) Immunostaining for ER (using mouse PDI antibody) and mitochondria (using rabbit TOM-20 antibody) of MNs derived from iPSC from patients carrying the P525L mutation and healthy controls with and without an overnight treatment with Tubastatin A (1 μM). The separate views show co-localized pixels in cell body and neurites. Scale bar = 5 μm.

(B) Quantification of stationary mitochondria, moving mitochondria and ratio between moving to total mitochondria normalized to a neurite length of 100 μm during 200 s from

MNs derived from patient and healthy control iPSC lines at the 4th week after plating with and without a Tubastatin A treatment. (n=10, n=14, n=11, n=13 for Ctr1+DMSO, Ctr1+Tubastatin A, P525L+DMSO and P525L+Tubastatin A respectively. Data are plotted as mean ± SEM; One-way ANOVA with post-hoc Tukey's test; **P values of 0.01 for t-test).

(C) Quantification of stationary ER vesicles, moving ER vesicles and ratio between moving to total vesicles normalized to a neurite length of 100 µm during 200 s from MNs derived from patient and healthy control iPSC lines at the 4th week after plating before and after Tubastatin A treatment. (n=10, n=16, n=12, n=14 for Ctr1+DMSO, Ctr1+Tubastatin A, P525L+DMSO and P525L+Tubastatin A respectively. Data are plotted as mean ± SEM; One-way ANOVA with post-hoc Tukey's test; *, **P values of 0.05 and 0.01 for t-test, respectively).

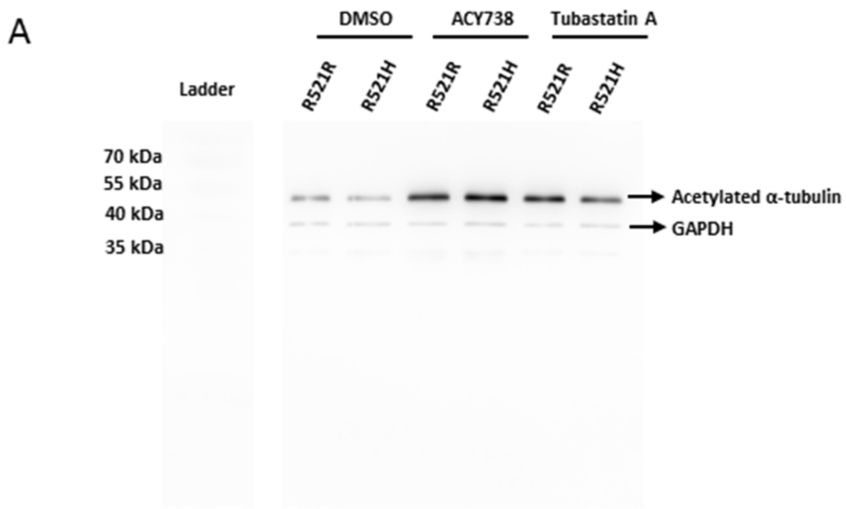

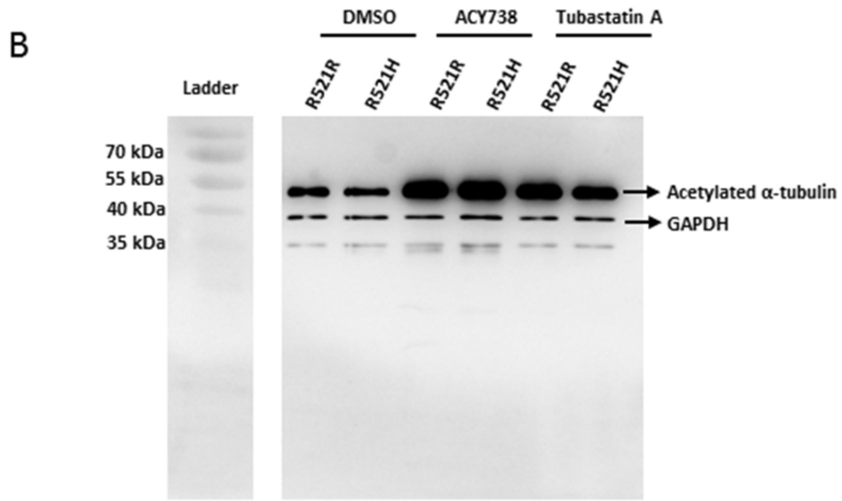

Supplementary Figure 10: Effect of HDAC6 inhibitors on acetylated α-tubulin

(A) Full version of the Western blot shown in Fig. 6D with the exposure used for acetylated α-tubulin

(B) Higher exposure of the same blot used for GAPDH in Fig. 6D.

Supplementary Table 1: Clinical information of patients and controls

Code	Diagnosis	Gender	Mutant	Age at biopsy	iPSC lines
**1/1**	FALS	F	R521H	33	1
**1/2**	FALS	M	R521H	presymptomatic	1
**2/2**	FALS	F	R521H	71	1
**3/1**	FALS	M	P525L	17	2
**3/2**	Healthy control1	F	No		1
**3/3**	Healthy control2	M	No		1
**4/1**	Healthy control3	M	No		1
**4/2**	Healthy control4	F	No		1

Supplementary Table 2: SNP analysis of iPSC

SNP label	fibroblasts	iPSC
	3/2 healthy control	3/2 healthy control
	allele1/allele2	allele1/allele2
C___1563023_10	FAM/FAM	FAM/FAM
C___1801627_20	VIC/VIC	VIC/VIC
C___2728408_10	FAM/FAM	FAM/FAM
C___1250735_20	VIC/VIC	VIC/VIC
C__15935210_10	VIC/FAM	VIC/FAM
C___7431888_10	VIC/FAM	VIC/FAM
C___3227711_10	VIC/VIC	VIC/VIC
C___1902433_10	VIC/VIC	VIC/VIC
C__30044763_10	FAM/FAM	FAM/FAM
C__31386842_10	FAM/FAM	FAM/FAM
C__33211212_10	VIC/FAM	VIC/FAM
C__26524789_10	FAM/FAM	FAM/FAM
C__11821218_10	FAM/FAM	FAM/FAM
C_____43852_10	VIC/VIC	VIC/VIC
C___1670459_10	FAM/FAM	FAM/FAM
C___8924366_10	VIC/VIC	VIC/VIC
C___1007630_10	VIC/VIC	VIC/VIC
C__11522992_10	FAM/FAM	FAM/FAM
C___7421900_10	VIC/FAM	VIC/FAM
C__10076371_10	VIC/FAM	VIC/FAM
C__26546714_10	VIC/FAM	VIC/FAM
C___1122315_10	VIC/VIC	VIC/VIC
C__27402849_10	VIC/FAM	VIC/FAM
C___7457509_10	VIC/FAM	VIC/FAM
C__29619553_10	VIC/VIC	VIC/VIC
C__11710129_10	FAM/FAM	FAM/FAM
C___2953330_10	VIC/FAM	VIC/FAM
C___1027548_20	NOAMP	UND
C___8850710_10	VIC/VIC	VIC/VIC
C___1083232_10	NOAMP	NOAMP
C__16205730_10	VIC/FAM	VIC/FAM
C___8938211_20	NOAMP	NOAMP

	fibroblasts	iPSC
SNP label	3/3 healthy control	3/3 healthy control
	allele1/allele2	allele1/allele2
C___1563023_10	VIC/FAM	VIC/FAM
C___1801627_20	VIC/FAM	VIC/FAM
C___2728408_10	VIC/FAM	VIC/FAM
C___1250735_20	FAM/FAM	FAM/FAM
C__15935210_10	VIC/FAM	VIC/FAM
C___7431888_10	VIC/FAM	VIC/FAM
C___3227711_10	VIC/FAM	VIC/FAM
C___1902433_10	VIC/FAM	VIC/FAM
C__30044763_10	FAM/FAM	FAM/FAM
C__31386842_10	FAM/FAM	FAM/FAM
C__33211212_10	FAM/FAM	FAM/FAM
C__26524789_10	FAM/FAM	FAM/FAM
C__11821218_10	VIC/VIC	VIC/VIC
C_____43852_10	FAM/FAM	FAM/FAM
C___1670459_10	VIC/FAM	VIC/FAM
C___8924366_10	VIC/FAM	VIC/FAM
C___1007630_10	VIC/VIC	VIC/VIC
C__11522992_10	FAM/FAM	FAM/FAM
C___7421900_10	VIC/FAM	VIC/FAM
C__10076371_10	VIC/VIC	VIC/VIC
C__26546714_10	VIC/VIC	VIC/VIC
C___1122315_10	VIC/FAM	VIC/FAM
C__27402849_10	VIC/FAM	VIC/FAM
C___7457509_10	VIC/FAM	VIC/FAM
C__29619553_10	VIC/FAM	VIC/FAM
C__11710129_10	VIC/FAM	VIC/FAM
C___2953330_10	VIC/FAM	VIC/FAM
C___1027548_20	VIC/VIC	VIC/VIC
C___8850710_10	VIC/FAM	VIC/FAM
C___1083232_10	VIC/VIC	VIC/VIC
C__16205730_10	FAM/FAM	FAM/FAM
C___8938211_20	FAM/FAM	FAM/FAM

	fibroblasts	iPSC
SNP label	1/1 patient(R521H)	1/1 patient(R521H)
	allele1/allele2	allele1/allele2
C___1563023_10	VIC/FAM	VIC/FAM
C___1801627_20	VIC/FAM	VIC/FAM
C___2728408_10	VIC/FAM	VIC/FAM
C___1250735_20	FAM/FAM	FAM/FAM
C__15935210_10	VIC/VIC	VIC/VIC
C___7431888_10	FAM/FAM	FAM/FAM
C___3227711_10	VIC/FAM	VIC/FAM
C___1902433_10	VIC/FAM	VIC/FAM
C__30044763_10	VIC/FAM	VIC/FAM
C__31386842_10	VIC/FAM	VIC/FAM
C__33211212_10	VIC/FAM	VIC/FAM
C__26524789_10	VIC/FAM	VIC/FAM
C__11821218_10	VIC/FAM	VIC/FAM
C_____43852_10	VIC/FAM	VIC/FAM
C___1670459_10	VIC/FAM	VIC/FAM
C___8924366_10	VIC/FAM	VIC/FAM
C___1007630_10	VIC/FAM	VIC/FAM
C__11522992_10	VIC/FAM	VIC/FAM
C___7421900_10	FAM/FAM	FAM/FAM
C__10076371_10	VIC/VIC	VIC/VIC
C__26546714_10	FAM/FAM	FAM/FAM
C___1122315_10	VIC/VIC	VIC/VIC
C__27402849_10	VIC/FAM	VIC/FAM
C___7457509_10	VIC/FAM	VIC/FAM
C__29619553_10	VIC/VIC	VIC/VIC
C__11710129_10	VIC/VIC	VIC/VIC
C___2953330_10	VIC/FAM	VIC/VIC
C___1027548_20	UND	UND
C___8850710_10	FAM/FAM	FAM/FAM
C___1083232_10	NOAMP	NOAMP
C__16205730_10	VIC/FAM	VIC/FAM
C___8938211_20	NOAMP	NOAMP

	fibroblasts	iPSC
SNP label	2/1 patient(R521H)	2/1 patient(R521H)
	allele1/allele2	allele1/allele2
C___1563023_10	VIC/VIC	VIC/VIC
C___1801627_20	VIC/VIC	VIC/VIC
C___2728408_10	VIC/VIC	VIC/VIC
C___1250735_20	VIC/FAM	VIC/FAM
C__15935210_10	VIC/VIC	VIC/VIC
C___7431888_10	FAM/FAM	FAM/FAM
C___3227711_10	FAM/FAM	FAM/FAM
C___1902433_10	FAM/FAM	FAM/FAM
C__30044763_10	VIC/FAM	VIC/FAM
C__31386842_10	VIC/FAM	VIC/FAM
C__33211212_10	NOAMP	VIC/FAM
C__26524789_10	VIC/FAM	VIC/FAM
C__11821218_10	FAM/FAM	FAM/FAM
C_____43852_10	VIC/FAM	VIC/FAM
C___1670459_10	VIC/FAM	VIC/FAM
C___8924366_10	VIC/FAM	VIC/FAM
C___1007630_10	FAM/FAM	FAM/FAM
C__11522992_10	NOAMP	VIC/FAM
C___7421900_10	VIC/FAM	VIC/FAM
C__10076371_10	VIC/VIC	VIC/VIC
C__26546714_10	NOAMP	VIC/FAM
C___1122315_10	VIC/VIC	VIC/VIC
C__27402849_10	VIC/FAM	VIC/FAM
C___7457509_10	VIC/VIC	VIC/VIC
C__29619553_10	VIC/VIC	VIC/VIC
C__11710129_10	VIC/FAM	VIC/FAM
C___2953330_10	VIC/FAM	VIC/FAM
C___1027548_20	UND	VIC/VIC
C___8850710_10	FAM/FAM	FAM/FAM
C___1083232_10	VIC/VIC	VIC/VIC
C__16205730_10	NOAMP	VIC/FAM
C___8938211_20	FAM/FAM	FAM/FAM

	fibroblasts	iPSC	isogenic control
SNP label	2/2 patient(R521H)	2/2 patient(R521H)	isogenic control(R521R)
	allele1/allele2	allele1/allele2	allele1/allele2
C___1563023_10	FAM/FAM	FAM/FAM	FAM/FAM
C___1801627_20	VIC/FAM	VIC/VIC	VIC/VIC
C___2728408_10	FAM/FAM	FAM/FAM	FAM/FAM
C___1250735_20	VIC/FAM	VIC/FAM	VIC/FAM
C__15935210_10	FAM/FAM	FAM/FAM	FAM/FAM
C___7431888_10	FAM/FAM	FAM/FAM	FAM/FAM
C___3227711_10	VIC/FAM	VIC/FAM	VIC/FAM
C___1902433_10	FAM/FAM	FAM/FAM	FAM/FAM
C__30044763_10	VIC/FAM	VIC/FAM	VIC/FAM
C__31386842_10	VIC/FAM	VIC/FAM	VIC/FAM
C__33211212_10	VIC/FAM	VIC/FAM	VIC/FAM
C__26524789_10	VIC/FAM	VIC/FAM	VIC/FAM
C__11821218_10	VIC/VIC	VIC/VIC	VIC/VIC
C_____43852_10	VIC/VIC	VIC/VIC	VIC/VIC
C___1670459_10	FAM/FAM	FAM/FAM	FAM/FAM
C___8924366_10	VIC/FAM	VIC/FAM	VIC/FAM
C___1007630_10	VIC/VIC	VIC/VIC	VIC/VIC
C__11522992_10	VIC/FAM	VIC/FAM	NOAMP
C___7421900_10	VIC/VIC	VIC/VIC	VIC/VIC
C__10076371_10	VIC/FAM	VIC/FAM	VIC/FAM
C__26546714_10	FAM/FAM	FAM/FAM	FAM/FAM
C___1122315_10	VIC/VIC	VIC/VIC	VIC/VIC
C__27402849_10	VIC/FAM	VIC/FAM	VIC/FAM
C___7457509_10	VIC/FAM	VIC/FAM	VIC/FAM
C__29619553_10	VIC/FAM	VIC/FAM	VIC/FAM
C__11710129_10	VIC/FAM	VIC/FAM	VIC/FAM
C___2953330_10	FAM/FAM	FAM/FAM	FAM/FAM
C___1027548_20	VIC/VIC	VIC/FAM	VIC/FAM
C___8850710_10	VIC/FAM	VIC/FAM	VIC/FAM
C___1083232_10	VIC/VIC	VIC/VIC	VIC/VIC
C__16205730_10	FAM/FAM	FAM/FAM	FAM/FAM
C___8938211_20	FAM/FAM	FAM/FAM	FAM/FAM

	fibroblasts	iPSC
SNP label	3/1 patient(P525L)	3/1 patient(P525L)
	allele1/allele2	allele1/allele2
C___1563023_10	VIC/FAM	VIC/FAM
C___1801627_20	VIC/FAM	VIC/FAM
C___2728408_10	FAM/FAM	FAM/FAM
C___1250735_20	VIC/FAM	VIC/FAM
C__15935210_10	VIC/FAM	VIC/FAM
C___7431888_10	VIC/VIC	VIC/VIC
C___3227711_10	VIC/VIC	VIC/VIC
C___1902433_10	VIC/FAM	VIC/FAM
C__30044763_10	FAM/FAM	FAM/FAM
C__31386842_10	FAM/FAM	FAM/FAM
C__33211212_10	FAM/FAM	FAM/FAM
C__26524789_10	FAM/FAM	FAM/FAM
C__11821218_10	VIC/FAM	VIC/FAM
C_____43852_10	VIC/FAM	VIC/FAM
C___1670459_10	FAM/FAM	FAM/FAM
C___8924366_10	VIC/VIC	VIC/VIC
C___1007630_10	VIC/VIC	VIC/VIC
C__11522992_10	FAM/FAM	FAM/FAM
C___7421900_10	VIC/FAM	VIC/FAM
C__10076371_10	VIC/VIC	VIC/VIC
C__26546714_10	VIC/FAM	VIC/FAM
C___1122315_10	VIC/VIC	VIC/VIC
C__27402849_10	VIC/FAM	VIC/FAM
C___7457509_10	VIC/FAM	VIC/FAM
C__29619553_10	VIC/FAM	VIC/FAM
C__11710129_10	VIC/FAM	VIC/FAM
C___2953330_10	VIC/FAM	VIC/FAM
C___1027548_20	VIC/FAM	VIC/FAM
C___8850710_10	VIC/FAM	VIC/FAM
C___1083232_10	VIC/VIC	VIC/VIC
C__16205730_10	FAM/FAM	FAM/FAM
C___8938211_20	FAM/FAM	FAM/FAM

Supplementary Table 3: Primers for qPCR, Semi-quantify PCR, sequencing, cloning related to Gene editing of hESCs by RMCE

Name	Forward (Fwd) Primer Sequence	Reverse (Rev) Primer Sequence
**Sendai**	5'TGCCCCAAGCAGACACCACCTG GCA 3'	
**Oct4**	5' GATGGCGTACTGTGGGCCC 3'	5' TGGGACTCCTCCGGGTTTTG 3'
**Nanog**	5' CAGCCCCGATTCTTCCAGTCCC 3'	5' CGGAAGATTCCCAGTCGGGTTCAC C 3'
**Sox2**	5' GGGAAATGGGAGGGGTGCAAAAG AGG 3'	5' TTGCGTGAGTGTGGATGGGATTGG TG 3'
**Rex1**	5' CAGATCCTAAACAGCTCGCAGAAT 3'	5' GCGTACGCAAATTAAAGTCCAGA 3'
**GAPDH**	5' ACCAGGAAATGAGCTTGACAAA 3'	5' TCAAGAAGGTGGTGAAGCAGG 3'
**Genomic *FUS* sequencing**	5' CATTTTGAGGGCTAGGTGGA 3'	5' AGTGAAAAGGGGGAAGAGGA 3'
**FUS cDNA**	5' AGTGGTGGCTATGAACCCAGAGGT 3'	5' AGTCATGACGTGATCCTTGGTCCC 3'
**3xFlag-FUS**	5' TGATTACAAGGATGACGATGACG 3'	5' TCCGTGGACTGGCTATAACC 3'
**FUS ORF Forward_AgeI**	5' ACCGACCGGTGGATGGCCTCAAAC GATTATACCC 3'	
**Wt FUS_Reverse_Mlu**		5' GCGTACGCGTTTAATACGGCCTCT CCCTGCGA 3'
**R521H FUS_Reverse_Mlu**		5' GCGTACGCGTTTAATACGGCCTCT CCCTGTGA 3'
**P525L FUS_Reverse_Mlu**		5' GCGTACGCGTTTAATACAGCCTCT CCCTGCGA 3'
**FUS Colony PCR**	5' TGGCTATGGACAGCAGGAC 3'	5' GGGTTCCTTCCGGTATTGT 3'
**Plasmid Sequencing1 (Fwd)**	5' AGGATGGGGCTTTTCTGTC 3'	
**Plasmid Sequencing2 (Fwd)**	5' TTTGATGACCCACCTTCAGC 3'	
**Plasmid Sequencing3 (Rev)**		5' GCTGAAGGTGGGTCATCAAA 3'
**Plasmid Sequencing4 (Rev)**		5' GTCCTGCTGTCCATAGCCA 3'

Supplementary Table 4: List of antibodies, Related to Immunocytochemistry and Western blotting

Antibody	Isotype	Dilution	Source
**SSEA4**	Mouse IgG	1/200	Santa Cruz
**Tra1-60**	Mouse IgG	1/1000	MIllipore
**Oct4**	Rabbit IgG	1/400	Santa Cruz
**Nanog**	Goat IgG	1/500	R&D
**Hb9**	Mouse IgG	1/100	DSHB
**Smi32**	Rabbit IgG	1/500	Abcam
**Isl1**	Rabbit IgG	1/200	MIllipore
**Tuj1**	Mouse IgG	1/500	Abcam
**ChaT**	Rabbit IgG	1/200	MIllipore
**Synapsin1**	Rabbit IgG	1/1000	MIllipore
**FUS**	Rabbit IgG	1/50	Proteintech
**NFL**	Goat IgG	1/500	Santa Cruz
**Tom20**	Rabbit IgG	1/500	Santa Cruz
**PDI**	Goat IgG	1/50	Santa Cruz
**Acetylated α-Tubulin**	Mouse IgG	1/5000	Sigma-Aldrich
**α-Tubulin**	Mouse IgG	1/5000	Sigma-Aldrich
**GAPDH**	Mouse IgG	1/5000	Ambion
